# Value of central review of RECIST v1.1 outcomes in the AGITG INTEGRATE randomised phase 2 international trial for advanced oesophago-gastric cancer

**DOI:** 10.1007/s00432-022-04404-4

**Published:** 2022-10-31

**Authors:** Katrin M. Sjoquist, Andrew Martin, Nick Pavlakis, David Goldstein, Eric Tsobanis, Daniel Moses, Richard Maher, Wendy Hague, Val Gebski, Martin R. Stockler, R. John Simes, the Australasian Gastro-Intestinal Trials Group (AGITG)

**Affiliations:** 1 NHMRC Clinical Trials Centre, University of Sydney, Sydney, Australia; 2 Cancer Care Centre, St George Hospital, Kogarah, Australia; 3 Department of Medical Oncology, Royal North Shore Hospital, Sydney, Australia; 4 Prince of Wales Hospital, Sydney, Australia; 5 Research Imaging NSW, UNSW, Sydney, Australia; 6 Radiology Department, Royal North Shore Hospital, Sydney, Australia

**Keywords:** Oesophageal cancer, Response evaluation criteria in solid tumours, Progression-free survival, Treatment effectiveness, Clinical trial

## Abstract

**Purpose:**

Activity estimates should be accurately evaluated in phase 2 clinical trials to ensure appropriate decisions about proceeding to phase 3 trials. RECIST v1.1. progression-free survival (PFS) is a common endpoint in oncology; however, it can be influenced by assessment criteria and trial design. We assessed the value of central adjudication of investigator-assessed PFS times of participants in a double-blind, randomised phase 2 trial evaluating regorafenib versus placebo in advanced gastro-oesophageal cancer (AGITG INTEGRATE) to inform plans for central review in future trials.

**Methods:**

We calculated the proportion of participants with a disagreement between the site investigator assessment and blinded independent central review and in whom central review resulted in a change, then evaluated the effect of central review on study conclusions by comparing hazard ratios (HRs) for PFS based on site review versus central review. Post-progression unblinding was assessed with similar methods. Simulation studies explored the effect of differential and non-differential measurement error on treatment effect estimation and study power.

**Results:**

Disagreements between site assessments versus central review occurred in 8/147 (5.4%) participants, 5 resulting in amended date of progression (3.4%). PFS HRs (sites vs central review progression dates) were similar (0.39 vs 0.40). RECIST progression occurred in 82/86 (95%) of cases where post-progression unblinding was requested by the site investigator.

**Conclusions:**

Blinded independent central review was feasible and supported the reliability of site assessments, trial results, and conclusions. Modelling showed that when treatment effects were large and outcome assessments blinded, central review was unlikely to affect conclusions.

## Introduction

Clinical trials require accurate endpoint assessment to yield valid and reliable estimates of treatment effects. This is particularly important in phase 2 trials with smaller numbers of participants, where variability in measurement and assessment has greater potential to affect trial outcomes. Accurate evaluation of treatment activity in phase 2 trials helps ensure decisions about moving to phase 3 evaluation are well informed (Dancey et al. 2009).

Overall survival (OS) is a gold standard for evaluating efficacy in phase 3 trials (Driscoll and Rixe 2009; Pazdur 2008). In oncology, progression-free survival (PFS) is often selected as the primary measure of treatment activity in phase 2 trials (Zhuang et al. 2009) as a surrogate for OS and other patient-centred endpoints (e.g. quality of life (Fiteni et al. 2014)). PFS is preferred to OS because it provides an earlier indication of activity, thereby reducing trial time and costs. Moreover, measures of disease stabilisation, such as PFS, are more appropriate endpoints of activity for targeted therapies that act by delaying tumour growth rather than reducing tumour volume (Stone et al. 2007), as might occur with cytotoxic chemotherapy. PFS has been accepted as a primary endpoint for regulatory approval when supported by other evidence of benefit, e.g. symptomatic improvement (Wilson et al. 2015).

The reliability of PFS as an endpoint is affected by the criteria used to assess it as well as aspects of trial design (Dancey et al. 2009; Bergmann et al. 2007). In oncology, assessments of progression are based on radiologic imaging of tumours according to the Response Evaluation Criteria in Solid Tumours (RECIST) v.1.1 (Eisenhauer et al. 2009). Application of RECIST is prone to variability in interpretation and other sources of measurement error. When measurement error of time-to-event endpoints occurs differently between treatment groups it can bias estimates of treatment effects and is considered differential error. Examples of this include more frequent imaging assessments in one group than another, leading to evaluation time bias, and investigators’ unblinded assessments of tumour size in open-label trials that are prone to earlier calls of progression in control group participants so that they can switch to a potentially active treatment.

Non-differential error is measurement error that is equally likely regardless of which treatment group participants have been allocated to. An example of this is differences between blinded central reviewers in applying RECIST that affects the observed date of progression. Non-differential error has little impact if the treatment effect is large (Korn et al. 2010). Differential error is of greater concern, as this can lead to over- or underestimation of treatment (Amit et al. 2010). Trial findings and decisions to proceed to a phase 3 trial may be affected when progression is deemed to occur earlier in the control group or later in the treatment group, or both, overestimating the magnitude of the treatment effect. Design considerations, e.g. use of placebo or blinding of outcome assessment can mitigate these potential biases (Bergmann et al. 2007; Stone et al. 2007). However, where these strategies are not possible, e.g. where treatment toxicities unmask treatment allocation, central review can identify, mitigate, and potentially quantify the magnitude of differential errors that could alter the estimated treatment effect (Amit et al. 2011).

Controversy exists about the required extent of blinded independent central review (BICR) of PFS. Given the time and expense required, the utility of reviewing 100% of cases has been questioned (Bergmann et al. 2007), with acceptance by some regulatory authorities that BICR may not be required in properly blinded trials (Amit et al. 2010). Although phase 3 trials require reliable assessment of outcomes, recommendations for phase 2 trials are less clear. A tailored independent central review approach considering design and endpoints is appropriate (Stone et al. 2011; Freidlin et al. 2007).

AGITG INTEGRATE was a randomised, double-blind, phase 2 trial evaluating the addition of regorafenib, a tyrosine kinase inhibitor of multiple angiogenic pathways, or placebo, to best supportive care in advanced gastro-oesophageal cancer (Pavlakis et al. 2016). PFS was chosen as the primary endpoint because regorafenib was anticipated to delay disease progression, rather than cause substantial tumour shrinkage. Effects on OS were expected to be closely correlated with effects on PFS, particularly in the absence of subsequent treatments to prolong survival. Treatment decisions were based on assessments of PFS by site investigators. Unblinding was allowed once RECIST progression occurred in participants suitable for further treatment.

Due to concerns that treatment allocation could be discernible in at least a proportion of participants as a result of the known toxicity profile of regorafenib (e.g. rash), and because unblinding of treatment allocation after documented progression (post-progression unblinding, PPU) was permitted in participants who were fit to receive open-label regorafenib subsequently, BICR of progression was planned a priori and conducted before analysing the trial outcome data. Our main objective was to compare BICR of PFS based on CT scan reports and clinical data versus standard site assessment of PFS and to determine the impact of discordance on the trial results and conclusions. In addition, we examined the effect of BICR of CT scan images (versus site assessment of the same) in a sample of participants; the potential for any bias in assessment where unblinding of treatment allocation had occurred; and the level of disagreement required to impact on trial conclusions in a simulation study.

## Methods

### Central review

Two separate processes were specified for BICR of PFS in INTEGRATE (Pavlakis et al. 2016). The first (study chairs’ review) evaluated de-identified CT reports or tumour measurement sheets for all participants against the investigator-determined date of progression by a central review team comprising the two study chairs and the clinical lead. Where there was agreement on the date of progression and choice of target lesions in both the site and central reviews, no further action was taken, and agreement noted. Where the reported information was insufficient, unclear, or disagreement existed, additional reports from other time-points were reviewed. Queries to site investigators were raised if the date of progression was still unable to be confirmed or disagreement remained. Subsequent review of de-identified CT scans or representative slices/screenshots were undertaken where additional information from queries to sites did not resolve discrepancies. The centrally adjudicated date of progression was used for the primary analysis where disagreements were unable to be resolved.

The second review process used independent radiologists blinded to treatment allocation to evaluate PFS in de-identified CT scans from a sample of participants. The first participant at each of 18 sites was identified at randomisation, and sites notified of the need to provide images for the complete set of scans at each time-point (baseline to progression or death without progression). Independent radiologists reported the scans in a two-step process. First, the radiologist reported on each set of scans without knowledge of the target/non-target lesions. Second, the same radiologist verified the accuracy of the measurements and application of RECIST v1.1 to the site-selected lesions. Where discrepancy between site and central review occurred, a second radiologist provided an independent review without knowledge of the conclusions of the first. For both processes, the reasons for disagreement were recorded.

A separate review process evaluated whether confirmation of RECIST progression occurred prior to PPU in these participants. Of the 152 participants, 86 (56%) were unblinded according to PPU processes, with 58% of those allocated to placebo subsequently receiving regorafenib. A second review team comprising the clinical leads not included in the study chairs’ review reviewed case records for all 86 participants after PPU. The results of each of the review components were not available to other review teams to prevent bias. The PPU date was compared to the date of radiologically confirmed progression to establish whether progression occurred prior to unblinding. The centrally confirmed date of progression was used where site and central investigators differed. Only the protocol criteria for RECIST v1.1 progression were reviewed centrally; there was no central review of other protocol criteria (e.g. performance status and organ dysfunction).

### Statistical methods

The rate of disagreement between site assessments and central assessments of PFS was calculated, and the sensitivity of analysis results to switching between site-assessed versus centrally assessed PFS was assessed by evaluating the magnitude of the change to the estimated hazard ratios (HRs).

A series of simulations were performed to explore what effect measurement error may have on a trial with similar characteristics. A set of 10,000 simulated trials were generated for a range of scenarios, which differed according to the specified magnitude of the underlying treatment effect and the type (non-differential/differential) and intensity of the measurement error imposed. Each simulated trial comprised 150 participants randomly allocated in a 2:1 ratio active drug (ACTIVE) versus placebo (PBO), who were followed until all observations of PFS were complete (i.e. no censored observations).

PFS times in each simulation were generated from an exponential distribution with a median of 4 weeks for the PBO group. The median PFS for the ACTIVE group was calculated by applying a series of hypothetical HRs to the PBO exponential distribution. Separate simulations used hypothetical HRs of 0.6, 0.67 or 0.75.

Non-differential measurement error was introduced into each simulation by shifting the true PFS time of a random proportion of *p* participants by 4 weeks. In separate simulations, *p* was set to 0, 5, 10, and 20%. The direction (forward or backward) of a given shift was chosen randomly with equal probability, and the impact of the error assessed in terms of its effect on statistical power (i.e. the probability of correctly rejecting the null hypothesis).

Differential measurement error was imposed by adding 4 weeks to the true PFS time for a random proportion (q) of participants in the ACTIVE group and subtracting 4 weeks from the true PFS time for a random proportion of *q* participants in the PBO group. Separate simulations set *q* to 0, 5, 10, and 20%. The bias associated with differential measurement error was assessed by reporting the mean observed HR for a given scenario relative to the true underlying HR.

## Results

### Study chairs’ review

Study chair review was performed for all 147 eligible participants. There were eight cases of disagreement between site assessment and study chair review (8/147, 5.4%; Table 1). Of these, five resulted in amendments by site investigators who concurred with the central review after answering queries. For the remaining three, site investigators were unable to substantiate their assessment of progression date and declined to amend their assessment. Analyses of the primary endpoint using the original site assessment dates for the 8/147 participants where this changed after central review did not affect the trial conclusions. The HR based solely on site assessment was 0.39 (95% CI 0.27–0.56) and very similar to that based on central review: 0.40 (95% CI 0.28–0.59).

**Table 1:** Disagreements between site assessment and study chair review

Participant	Reason for disagreement	Date of PD/PFS (site)	Date of PD/PFS (central)	Detail/response
009	Lesion data entered did not support progression or correlate with CT report	9 May 2013	9 May 2013	Site agreed with central after resolving queries
013	Ad hoc scan demonstrated new lesions prior to CT scheduled by protocol	18 March 2013	5 March 2013	Site agreed with central after queries
023	Inconsistencies between tumour measurements entered and CT reports due to choice of different target lesions	19 July 2013	9 June 2013	Site agreed with central after resolving queries
057	Site indicated SD 5 Aug 13 prior to EOT on 19 Aug 13 due to AE. New liver meds described on CT report and confirmed on review of images	19 August 2013 SD at EOT/withdrawal	5 August 2013	Site disagreed/could not substantiate their assessment
063	Ad hoc CT identified new lesions consistent with progression	12 November 2013	6 November 2013	Site agreed with central after resolving queries
085	Site indicated PD based on new lesion. On central review lesion appeared to be present at baseline	22 October 2013	SD on 22 October 2013. PD date unable to be determined as no further scans prior to death 14 Nov 2013	Site agreed with central after queries
110	Site indicated PD based on new lesion. On central review lesion appeared to be present at baseline	14 January 2014	18 November 2013	Site disagreed/could not substantiate their assessment
129	Insufficient evidence from reports or data entered to support PD. Participant unblinded by site	11 March 2014	Participant unblinded by site. Censored at 11 March 2014 with SD	Site disagreed/could not substantiate their assessment

### Independent radiologists’ review

A total of 18 participants were selected for BICR of CT images by independent radiologists, with another 2 added after study chair review. Central radiologist and site assessments ultimately agreed in 18 of the 20 participants. In the two cases with unresolved disagreements, each of the radiologists reached different conclusions on initial review: one agreed with the site assessment, the other with the study chairs’ assessment.

Of the 18 cases where there was eventual agreement between site and radiology review, 6 underwent review by a second radiologist because the first disagreed with the site conclusions. In four of these six cases, the second radiologist agreed with the site assessment and in the remaining two, with the first radiologist. Following a secondary review where radiologists were provided with information regarding details of the target lesions chosen by site investigators, the conflict was resolved in all but two cases (Table 2).

**Table 2:** Cases with unresolved disagreements between central and site assessments following independent radiologist review

Participant	Site-assessed progression date	Radiology review (1)	Radiology review (2)	Reason for disagreement	Significance
013	18 Mar 2013	SD at 18 Mar 13*	PD at 5 Mar 13*	Significance of new ascites	Disagreement in interpretation of RECIST
062	4 Sep 2013	SD at 4 Sep 13	PD at 4 Sep 13	Different choice of TLs; subsequent review against site chosen lesions agreed with site	Issue with application of RECIST/choice of target lesions

^*^An ad hoc scan was performed on 5 March prior to scheduled assessment on 18 March. This identified new ascites. This was considered a new lesion consistent with PD by one of the radiologists

### PPU review

Site investigators requested unblinding of treatment allocation for 86/152 (57%) participants prior to the initial analysis cutoff date: 50 assigned regorafenib and 36 assigned placebo. Globally, the PPU review found evidence to support progressive disease having been documented prior to requesting PPU in 82/86 (95%) of cases (Table 3). Of the four discrepant cases, in two the PPU results were supported by the review, with no evidence of progressive disease prior to the date of unblinding; one participant ended treatment for “clinical progression” and one for “clinician preference” with no evidence of radiologic progression of disease prior to PPU.

**Table 3:** PPU review

Region	Randomised participants (*n* =)	PPU’s participants* *n* (%)	Results of central PPU review	
			Unblinded after PD confirmed *n* = (%)	Unblinded after treatment ended—other reason *n* = (%)
ANZ	81	46 (57)	42 (91)	4 (9)
Korea	54	29 (54)	29 (100)	0 (0)
Canada	17	11 (65)	11 (100)	0 (0)
Global	152	86 (57)	82 (95)	4 (5)

### Simulation of the effect of measurement error

The results of the simulation studies are presented in Tables 4 and 5. For the scenarios explored, every 10% increase in the prevalence of differential error resulted in a decrease of 0.05–0.07 in the observed HR, indicating a greater treatment effect. Study power decreased on average by 0.03–0.04 for every 10% increase in the rate of non-differential error.

**Table 4:** Mean observed HRs assuming differential errors in assessment of PFS

True HR	Prevalence of differential errors in assessment of PFS			
	0%	5%	10%	20%
0.60	0.60	0.57	0.54	0.49
0.67	0.66	0.63	0.60	0.54
0.75	0.75	0.71	0.67	0. 60

**Table 5:** Power to reject the null hypothesis of no treatment effect assuming differential errors in assessment of PFS

True HR	Prevalence of non-differential errors in assessment of PFS			
	0%	5%	10%	20%
0.60	0.84	0.81	0.80	0.76
0.67	0.63	0.61	0.58	0.55
0.75	0.38	0.36	0.34	0.32

#### Discussion

The choice of PFS as the primary endpoint for the INTEGRATE trial was based on the phase of development and the treatment setting. However, the use of PFS rendered the trial vulnerable to two important sources of bias. First, cross-over was allowed after progression. This had the potential to encourage overcalling of progression for subjects suspected to be on placebo based on rapid progression and an absence of obvious treatment-related toxicities. Second, site investigator assessments of PFS were used as the primary endpoint. In addition, the challenges in identifying RECIST target lesions in advanced gastric cancer patients with predominantly intra-abdominal disease, often peritoneal and associated with ascites, complicates the assessment of progression, although this would be expected to contribute mainly to non-differential error if allocation concealment was effective.

In INTEGRATE, concerns existed that treatment toxicities might unblind assessors. In trials where blinding is not done or may be unreliable, BICR is often recommended and has the potential to quantify the reliability of results and identify types and frequencies of error (Walovitch et al. 2013). Real-time BICR of imaging was not considered feasible for this academic, investigator-initiated international cooperative group trial, as full BICR is expensive and may exaggerate treatment effects on PFS through informative censoring (Stone et al. 2018).

Strategies for limited BICR have been proposed for randomised phase 3 trials (Amit et al. 2011; Dodd et al. 2008, 2011; Stone et al. 2015). Two different audit methods were evaluated by Zhang and colleagues (2013), who concluded that BICR audit of a random sample is a viable alternative to full BICR. These and other authors have noted that the selection of audit strategy may need to be determined on a case-by-case basis (Stone et al. 2011; Freidlin et al. 2007). Such strategies have not previously been formally evaluated in phase 2 trials. On these considerations, we included a review of radiology scans on a sample of participants; this indicated that RECIST 1.1 was applied appropriately, and that review of more cases was unlikely to change the conclusions.

Our review of eCRFs, CT reports and other documents from the INTEGRATE trial demonstrated a small number of discrepancies (8/147, 5.4%) that had no material effect on the observed treatment effect on PFS or trial conclusions. Central review of a sample of CT scans demonstrated that discrepancies with sites were mainly due to either the choice of different target lesions at baseline, which should not lead to differential (biased) assessment of response, or different interpretations or incorrect application of RECIST 1.1 by some site investigators. The majority of discrepancies observed did not affect the results or conclusions and were due to known ambiguities in tumour assessment, e.g. the interpretation of new or increasing ascites. Had this review indicated that RECIST criteria were not being appropriately applied, review of imaging in a larger proportion of participants would have been performed.

The main limitation of this study was that central review was confined to imaging reports and tumour assessment worksheets for most participants. Our central reviewers were blinded, and this will not detect biases due to a site investigator’s knowledge or suspicion of treatment allocation through awareness of treatment side effects. Our simulations suggest that when treatment effects are large and/or imaging is reported by blinded assessors, consistent and large biases would be needed to influence the results (Korn et al. 2010). Similarly, given the substantial treatment effect in this trial (HR 0.40; 95% CI 0.28–0.59; *P* < 0.001), a large amount of error would need to exist to change trial conclusions.

In a blinded trial, most disagreements in tumour assessments are likely to be non-differential, and hence unlikely to be of consequence. The main area where biased assessment might occur is in participants for whom treatment allocation has been unblinded. In INTEGRATE, an assessment of progressive disease was required prior to unblinding, and whenever this was not confirmed or changed after unblinding, a central review of the actual images was done. The PPU review was undertaken to evaluate whether systematic (differential) bias was likely to affect results as a consequence of assessment bias due to actual or suspected unblinding based on a lack of treatment-related toxicity in rapidly progressing participants. This was found to be of limited consequence in this trial, with most participants having evidence of radiologic progression prior to unblinding. Our simulations indicate that in trials with a large treatment effect, a large degree of systematic non-random error would be necessary to affect trial outcomes and conclusions.

Undertaking central review using the process we have described may be an appropriate method to quantify the extent of potential error in similarly designed trials. While the value of this process may be limited if effect sizes are large, such reviews should be performed prior to knowledge of study outcomes. In INTEGRATE, we demonstrated that most of the discrepancies detected resulted from incorrect application or interpretation of RECIST 1.1. by site investigators, with a smaller proportion occurring due to differences in interpretation. Treatment allocation was unblinded in few participants without prior evidence of disease progression, indicating a low risk of differential error.

In conclusion, our rigorous approach to outcome assessment provides reassurance about conclusions based on PFS in INTEGRATE and provides a model that could be applied to similar placebo-controlled trials. In trials where allocation concealment is effective, central review may not be necessary where effect sizes are substantial. The value of a similar process in open-label trials is outside the scope of this work, but worthy of future exploration.

## Data Availability

The data underlying this article will be shared on reasonable request to the corresponding author.

## References

[CR1] Amit O, Bushnell W, Dodd L, Roach N, Sargent D (2010). Blinded independent central review of the progression-free survival endpoint. Oncologist.

[CR2] Amit O, Mannino F, Stone AM, Bushnell W, Denne J, Helterbrand J, Burger HU (2011). Blinded independent central review of progression in cancer clinical trials: results from a meta-analysis. Eur J Cancer.

[CR3] Bergmann L, Hirschfeld S, Morris C, Palmeri S, Stone A (2007). Progression-free survival as an end-point in clinical trials of biotherapeutic agents. Eur J Cancer (suppl).

[CR4] Dancey JE, Dodd LE, Ford R, Kaplan R, Mooney M, Rubinstein L, Schwartz LH, Shankar L, Therasse P (2009). Recommendations for the assessment of progression in randomised cancer treatment trials. Eur J Cancer.

[CR5] Dodd LE, Korn EL, Freidlin B, Jaffe CC, Rubinstein LV, Dancey J, Mooney MM (2008). Blinded independent central review of progression-free survival in phase III clinical trials: important design element or unnecessary expense?. J Clin Oncol.

[CR6] Dodd LE, Korn EL, Freidlin B, Gray R, Bhattacharya S (2011). An audit strategy for progression-free survival. Biometrics.

[CR7] Driscoll JJ, Rixe O (2009). Overall survival: still the gold standard: why overall survival remains the definitive end point in cancer clinical trials. Cancer J.

[CR8] Eisenhauer EA, Therasse P, Bogaerts J, Schwartz LH, Sargent D, Ford R, Dancey J, Arbuck S, Gwyther S, Mooney M, Rubinstein L, Shankar L, Dodd L, Kaplan R, Lacombe D, Verweij J (2009). New response evaluation criteria in solid tumours: revised RECIST guideline (version 1.1). Eur J Cancer.

[CR9] Fiteni F, Westeel V, Pivot X, Borg C, Vernerey D, Bonnetain F (2014). Endpoints in cancer clinical trials. J Visc Surg.

[CR10] Freidlin B, Korn EL, Hunsberger S, Gray R, Saxman S, Zujewski JA (2007). Proposal for the use of progression-free survival in unblinded randomized trials. J Clin Oncol.

[CR11] Korn EL, Dodd LE, Freidlin B (2010). Measurement error in the timing of events: effect on survival analyses in randomized clinical trials. Clin Trials.

[CR12] Pavlakis N, Sjoquist KM, Martin AJ, Tsobanis E, Yip S, Kang YK, Bang YJ, Alcindor T, O'Callaghan CJ, Burnell MJ, Tebbutt NC, Rha SY, Lee J, Cho JY, Lipton LR, Wong M, Strickland A, Kim JW, Zalcberg JR, Simes J, Goldstein D (2016). Regorafenib for the treatment of advanced gastric cancer (INTEGRATE): a multinational placebo-controlled phase II trial. J Clin Oncol.

[CR13] Pazdur R (2008). Endpoints for assessing drug activity in clinical trials. Oncologist.

[CR14] Stone A, Wheeler C, Carroll K, Barge A (2007). Optimizing randomized phase II trials assessing tumor progression. Contemp Clin Trials.

[CR15] Stone AM, Bushnell W, Denne J, Sargent DJ, Amit O, Chen C, Bailey-Iacona R, Helterbrand J, Williams G, PhRMA Working Group (2011). Research outcomes and recommendations for the assessment of progression in cancer clinical trials from a PhRMA working group. Eur J Cancer.

[CR16] Stone A, Macpherson E, Smith A, Jennison C (2015). Model free audit methodology for bias evaluation of tumour progression in oncology. Pharm Stat.

[CR17] Stone A, Gebski V, Davidson R, Bloomfield R, Bartlett J, Sabin A (2018). Exaggeration of PFS by blinded, independent, central review (BICR). Ann Oncol.

[CR18] Walovitch RC, Yao B, Chokron P, Le H, Bubley G (2013). Subjective endpoints in clinical trials: the case for blinded independent central review. Open Access J Clin Trials.

[CR19] Wilson MK, Karakasis K, Oza AM (2015). Outcomes and endpoints in trials of cancer treatment: the past, present, and future. Lancet Oncol.

[CR20] Zhang JJ, Zhang L, Chen H, Murgo AJ, Dodd LE, Pazdur R, Sridhara R (2013). Assessment of audit methodologies for bias evaluation of tumor progression in oncology clinical trials. Clin Cancer Res.

[CR21] Zhuang SH, Xiu L, Elsayed YA (2009). Overall survival: a gold standard in search of a surrogate: the value of progression-free survival and time to progression as end points of drug efficacy. Cancer J.

